# News and Notes

**Published:** 1902-07

**Authors:** 


### NEWS AND NOTES.

(We are indebted to the *Medical Age* for many of these notes.)

---

THE *Atlanta Medical and Surgical Journal* and *Southern Medical Record* having been consolidated into THE ATLANTA JOURNAL-RECORD OF MEDICINE, exchanges will please be sent only to the latter. We are getting many duplicates.

---

ALL the vacancies in the Navy Medical Service are filled for the first time since the civil war.

---

THE Texas State Medical Association has adopted resolutions advocating State control of tuberculosis.

---

PROFESSOR VIRCHOW has so far recovered from his recent accident as to be able to leave Berlin for the seashore.

---

ACCORDING to statistics the number of female physicians throughout the entire world is about 8,000, two-thirds of whom live in America.

---

THE bill to permit the retirement of Surgeon-General Sternberg with the rank of major-general was defeated in the House of Representatives.

---

THE Army Medical Board, which has been in session in Washington since April 3, for the examination of candidates for commission in the medical department, has examined six classes, yet has found but nine candidates qualified for appointment.

---

FROM bacteriological investigations made at the Hospital du Bey, Algiers, it has been found that almost all varieties of bacteria occur upon currency. Metal, however, has a certain antiseptic effect, causing the bacteria to be short-lived. The action of gold is less marked in this respect than in other metals. Typhoid bacilli will live from five to seven days on a gold piece, but die in less than eighteen hours on other metals.—*Philadelphia Medical Journal.*

---

AN assistant coroner of New York recently reported some young internes at Bellevue to the superintendent for negligence about calling the coroners to take ante-mortem statements. It is said that the criticisms of the coroner's assistant were so spicy that the internes have since that time used every pretext for calling the coroners to the hospital, and that the coroners are good and tired.

---

THE Martinique disaster has reduced a good many medical students from that island to a state of destitution in Paris. According to *Progrès Medical* an effort is being made to aid these unfortunate young men. Two committees have been formed—one to secure the remission of their school expenses, and another to make up for them, as far as possible, their allowances for support. The appeal in this matter is specially addressed to medical men.

---

DR. WILFRID GRENFELL, the English physician who is devoting his life to the amelioration of the fishermen of Labrador, writes that at present there is great destitution among the inhabitants. His work ranges from treating a compound fracture to making a pair of boots from the skin of a freshly killed seal. In the summer Dr. Grenfell journeys along the coast in a steamer, the hospital ship Strathcona; in winter his tours are made on dog sleds. He has recently established a third hospital at St. Anthony, on the north French shore.

NEXT to Sardinia, the province of Basilicata, near Naples, is the largest malarious tract in Italy. In killing the larvæ of mosquitoes an aniline dye is used, which is efficient even when diluted to the extent of 0.00031 per 1000. A powder is also used, made from the flowers of the *Pyrethrum roseum*, cultivated near Ceprano. Valerian root mixed with this renders it more efficacious. Professor Grassi, following experiments with a compound called esanofele, composed of quinine, arsenic, iron and bitter herbs, six pills of which were given daily, speaks highly of his results.—*Exchange*.

---

THE new plan of organization of the American Medical Association seems to have worked well. The House of Delegates proved to be a very serviceable body and far less unwieldy than the general sessions. To be a member of the House of Delegates is, however, to forego section meetings almost wholly. Dr. Wm. H. Welch and Dr. Wm. Osler represented Maryland in the House of Delegates. The next meeting of the American Medical Association will be held at New Orleans in May, 1903. Among the interesting things done by the House of Delegates was a resolution offered by Drs. Forshay and McCormack, and adopted with enthusiasm. The resolution expressed the sense of the House of Delegates that "the solicitation of votes for office is not in keeping with the dignity of the medical profession, nor in harmony with the spirit of the Association, and that such solicitation should be taken to be a disqualification for any office in the gift of the Association."

---

AT a recent meeting of the Academy of Medicine of Paris MM. Chipault and Lefur read the clinical history of an interesting case of a man who, without any appreciable affection of either the nervous system or of his viscera, suffered atrocious abdominal pains for years. During these paroxysms of pain, which were most intense at the naval, the body was strongly flexed, and the abdominal muscles contracted violently. The hyperalgesic region corresponded to the area of distribution of the eighth, ninth, and tenth dorsal ribs. Hypodermic injections having given no good results, spinal injections were tried, and they also failed. It was now decided to cut the intercostal nerves of the area of greatest pain, and this was done without any benefit. The posterior laminæ of the eighth, ninth, and tenth dorsal vertebræ were then incised, and the posterior roots of the corresponding spinal nerves cut. When the spinal cord was exposed there was found a localized patch of arachnitis of some standing. The result was eminently satisfactory. At the end of the month the hyperalgesic zone had disappeared, and gradually the severity of the paroxysms of pain diminished until they finally ceased.—*Medical Press.*

---

A NUMBER of cases of leprosy have been found among the natives of the Island of Guam, and steps have been taken, says the *Medical Record,* by the medical authorities of the navy for their collection and segregation. The establishment of a permanent colony on thirty acres of ground on Tumon Bay Beach, within practicable reach of Agana, the chief town of Guam, by a fairly good road, is intended. Steps have already been taken to acquire this ground, and plans are being prepared for the necessary buildings. It is understood that the nursing of the unfortunates will be undertaken by a Catholic religious organization. The report says: "It appears that during the interregnum immediately following the withdrawal of the Spanish government from the island, the lepers gradually slipped out of the Asan hospital, being received and cared for by the kind-hearted people of the villages, who are loath to betray them. When the last inmate died it was generally asserted that the disease was practically extinct; several reported cases proved, upon examination, not to be leprosy, and such search as was feasible failed to discover any until now. It is believed that no material harm in the way of dissemination has resulted from this recent lack of segregation."

